# The deviation-from-familiarity effect: Expertise increases uncanniness of deviating exemplars

**DOI:** 10.1371/journal.pone.0273861

**Published:** 2022-09-01

**Authors:** Alexander Diel, Michael Lewis

**Affiliations:** School of Psychology, Cardiff University, Cardiff, United Kingdom; University of Sydney, AUSTRALIA

## Abstract

Humanlike entities deviating from the norm of human appearance are perceived as strange or uncanny. Explanations for the eeriness of deviating humanlike entities include ideas specific to human or animal stimuli like mate selection, avoidance of threat or disease, or dehumanization; however, deviation from highly familiar categories may provide a better explanation. Here it is tested whether experts and novices in a novel (greeble) category show different patterns of abnormality, attractiveness, and uncanniness responses to distorted and averaged greebles. Greeble-trained participants assessed the abnormality, attractiveness, uncanniness of normal, averaged, and distorted greebles and their responses were compared to participants who had not previously seen greebles. The data show that distorted greebles were more uncanny than normal greebles only in the training condition, and distorted greebles were more uncanny in the training compared to the control condition. In addition, averaged greebles were not more attractive than normal greebles regardless of condition. The results suggest uncanniness is elicited by deviations from stimulus categories of expertise rather than being a purely biological human- or animal-specific response.

## Introduction

Artificial entities that are close to but deviate from the norm of human appearance tend to reap negative appraisal like uncanniness [1–3]. Uncanniness is more sensitive to structural deviations in highly realistic compared to unrealistic faces [4, 5], or to distortions in human compared to animal faces [6, 7]. While evolutionary mechanisms or processes specific to human or animal stimuli, like avoidance of indicators of disease [8], psychopathy [9], or dehumanization [10], have been proposed as explanations, sensitivity to subtle configural distortions in highly familiarized categories like faces may also be increased due to the high-degree familiarity or expertise with human face configurations [4–8, 11]; However, the effect of expertise of familiarity manipulation of the uncanniness of deviating exemplars has not yet been tested. If the hypotheses were true, deviating exemplars of any stimulus category would be potentially uncanny given sufficient expertise. This *deviation-from-familiarity effect* is tested here by exploring the uncanniness of configural distortions in novel objects (greebles) with or without acquired expertise.

Below, the common properties of faces and greebles are explained, followed by a discussion of attractiveness of faces based on prototypicality and its relation to attractiveness of greebles for greeble experts. This is followed by a discussion of the uncanny valley and how assessment of uncanniness in objects by either experts or novices on those objects can further our understanding of the uncanny valley. The subsequent experiment tests conflicting accounts of the causes of uncanniness.

### Face processing and greeble expertise

Human faces usually carry similar structural features like the number and positions of eyes, noses, and mouth. Faces however differ in their relative information of these features. Thus, differentiation and identification of faces focusses on relational information, a process called a configural processing [12, 13].

Some researchers have argued that configural processing can occur for non-face categories [14] like bodies [15, 16] and biological motion [17, 18]. Furthermore, a configural or holistic processing style has been observed for stimulus categories when participants are experts in those categories, like experts for animals [19] and fingerprints [20, 21]. Perceptual expertise develops through enhanced experience in differentiating individual exemplars of a stimulus category which would naturally occur for conspecific faces [22–25]. Furthermore, expertise can be manipulated by training participants to differentiate individual objects of a category of novel, computer-generated stimuli like *greebles* [26]. Greeble-trained participants show cognitive performance and neurophysiological activity for greebles comparable to those observed for faces [27–29]. Thus, expertise training approximates cognitive and neurophysiological processing styles typically observed for faces and may be used to investigate the effect of expertise on face-related processing.

### The uncanny valley and face uncanniness

The uncanny valley effect predicts negative evaluation of near humanlike artificial entities like androids or computer-generated characters [2, 8]. The cognitive mechanisms underlying the effect remain unclear. Previous research has shown that structurally distorted faces elicit an uncanny valley effect, and higher levels of realism also decrease the range of acceptable faces and the sensitivity to facial distortions [4, 5, 30].

The affective or perceptual component of the uncanny valley has been described as a specific sensational response related to eeriness, creepiness, strangeness, and coldness [2, 30–33]. Whereas a variety of measures and interpretations of the uncanny valley’s affective component exist, a recent meta-analysis on the uncanny valley’s methodology suggests that specific anxiety-related semantic items like *eerie*, *creepy*, and *uncanny*, or anomaly-related items like *strange* and *weird* to be effective measures to capture the effect [31]. This study will focus on the experiences of uncanniness and abnormality and their proposed causes.

Some explanations of the uncanny valley propose evolutionary origins [6, 8, 10]. Mate selection theories propose that uncanniness of deviating features is caused by an evolutionary mechanism to avoid potential mates with bad fitness, and threat avoidance theory proposes that the uncanny valley is part of a mechanism to detect and avoid indicators of contagious disease [8]. Other theories of the uncanny valley focus on the perception of mind or animacy,or lack thereof [34–36], the detection of possibly dangerous (malevolent) intention in another human actor [9], or dehumanization of near humanlike entities [10, 37]. Thus, several theories presuppose human (or animal) specificity of the uncanny valley.

An uncanny valley is also observed when using animal stimuli [38, 39], but the sensitivity of the effect to facial distortions remains stronger in human faces compared to animal faces [6]. Furthermore, recent evidence shows that the effect of facial distortion on uncanniness ratings is enhanced when faces are upright (compared to inverted) and familiar (compared to novel), and that this effect is mediated by perceptual sensitivity to detect facial distortions [40]. Thus, sensitivity to uncanniness ratings is enhanced for deviations of exemplars of personally familiar stimulus categories like upright and familiar faces, possibly through an enhanced perceptual sensitivity to deviations.

Because the sensitivity of uncanniness to distortions is enhanced for familiar (experience with various exemplars of a category) stimulus categories, some researchers have suggested that the degree of *expertise* (the ability to recognize and differentiate individual exemplars within a category) with a category enhances the uncanny valley sensitivity, possibly by strengthening the degree of configural processing, increasing the sensitivity to even slight configural distortions [4–7, 11, 40]. While some researchers have proposed that cognitive disfluency underlies the uncanny valley, possibly caused by categorization difficulty [41–43], categorization confusion or difficulty and uncanniness ratings follow different trajectories across a range of stimuli varying on the degree of human likeness [1, 44]. Furthermore, some researchers have argued that general cognitive theories like disfluency or dissonance are insufficient in explaining the uncanny valley as they have not been related to specific sensations of eeriness or uncanniness in previous research [7, 31].

Rather than uncanny stimuli being intrinsically uncanny, uncanniness may instead result from stimuli that deviate from an experienced or learnt norm, modulated by the degree of familiarity or expertise with the stimulus category. Such a *deviation-from-familiarity effect* would occur for any type of stimulus that strongly deviates from a familiar configural pattern–that is, the same pattern underlying all category exemplars. Such an expertise-based theory would explain a wide range of observations in uncanny valley research like the effect of realism on sensitivity to uncanniness [5, 30], human compared to animal faces [6], or upright compared to inverted faces [40], and would furthermore propose a testable universality of the uncanny valley across different stimulus categories given sufficient perceptual expertise. While familiarity and expertise refer to two different concepts, expertise requires sufficient familiarity. Hence, expertise is here used as a marker of familiarity.

The effect of expertise on uncanniness ratings has not yet been actively investigated. Greeble stimuli have been successfully used to train expertise and are designed to be individually recognizable based on differences in single features that follow the same configural pattern. Thus, greeble expertise training is a viable candidate method to investigate the effect of prolonged exposure on the uncanniness of configural distortion. Thus, the present research will focus on the cognitive causes of uncanniness as predicted by previous theories on the uncanny valley. An uncanny valley function is not replicated here, nor are humanlike stimuli used. Nevertheless, the results may provide important insights into the cognitive mechanisms underlying the uncanny valley phenomenon.

### Preference of prototypicality

The variety of facial structure can be represented by a multidimensional face space where each point in the space represents an individual face based on its structure along multiple dimensions [45, 46]. The most averaged faces are stored at the face space centre while increasing the distance from the centre increases facial distinctiveness. Averaged (composite) faces are perceived as more attractive than normal faces, and individual faces closer to their group prototype (e.g., sex or ethnicity) are more attractive than normal faces [47, 48]. Caricatured composite faces of attractive faces are more attractive than full composite faces [49], challenging the notion that averageness itself is the attractive feature. Instead, averaging faces may enhance evolutionary signals of fertility and genetic fitness [50].

The effect of averaging on attractiveness has however also been observed for schematic depictions of animal and object categories and increasing the number of stimuli used to create the composite enhances attractiveness [51, 52], suggesting that a general cognitive process underlies the attractiveness of averaged stimuli. Some researchers argue that prototypical stimuli in general are perceived as more attractive (*prototypicality* or *beauty-in-averageness effect*; [52, 53]), likely driven by an intrinsic preference for stimuli that are processed fluently (*cognitive fluency*; [54, 55]). *Prototypes* are exemplars that are the best representatives of a category [56], and multiple studies have shown an aesthetic preference (e.g., higher attractiveness or beauty ratings) for exemplars that are closer to the prototypes compared to more distinct exemplars [56–58]. Thus, prototypicality itself should also increase facial attractiveness. Since the variance of facial attractiveness decreases with inversion [59–61], configural information is likely used when assessing facial aesthetics, likely by enhancing the ability to detect closeness to (and deviations from) face prototypes [40].

The effect of active expertise manipulation on the *prototypicality effect* has not yet been tested. If the beauty-in-averageness theory were true then increasing expertise with a novel stimulus category would increase the aesthetic appeal of averaged exemplars, even if the averaged exemplars would not have been viewed before. Greeble training is thus an adequate method to test the effect of expertise on the *prototypicality effect*.

### Research question and hypotheses

The present work will investigate cognitive causes of experiences of uncanniness and abnormality. It is proposed that aesthetic judgment of exemplars based on their distance to a category’s centre or prototype should depend on the degree of perceptual expertise with the category. Exemplars distant from the “normal range” of observed exemplars surrounding the centre should be rated as more uncanny, while stimuli closer to the centre should appear more attractive. Thus, greeble expertise training should increase the attractiveness of averaged greebles relative to normal greebles, while increasing the relative uncanniness of configurally distorted greebles deviating from the norm.

The current study is the first to investigate the effect of expertise training on these two phenomena: The uncanniness of distorted category exemplars and the attractiveness of averaged (prototypical or blended) category exemplars. Participants rated *uncanniness* and *attractiveness* of normal, averaged, and distorted greebles either after 5-day expertise training (*training condition*) or without expertise training (*control condition*).

The following hypotheses were tested. First, distorted greebles are rated as more *uncanny* after training (*training condition*) than distorted greebles without training (*control condition*) when compared to the normal greebles.

Second, without training, normal and averaged greebles do not differ in *attractive* ratings but with training, normal greebles are rated significantly less *attractive* than averaged greebles.

## Methods

### Participants

Participants were 45 Cardiff University psychology students randomly split into 21 participants in a *training group* and 24 in a *control group*. Participants had a mean age of *M*_*age*_ = 19.52, *SD*_*age*_ = 1.42, and 36 were female. Because the interpretation of the results was predominantly based on Bayesian inference which is not affected by sample size, sample size was decided on the Bayesian stopping rule after collecting an initial set of participants, and because evidence either in favor or against the null hypothesis was already present with the initial sample size, data collection was stopped at that point [62, 63]. For p-value statistics, a post-hoc power analysis revealed that a power of 1- β = 0.8 would be achieved with the given sample size and an effect size of *d* = 0.4.

### Stimuli

#### Greeble training set

A set of 30 asymmetrical greeble stimuli from the tarrlab stimulus database (see https://sites.google.com/andrew.cmu.edu/tarrlab/stimuli) were used for the study. The greeble set consisted of six individual greebles per five families. Greeble families differed by having distinct body shapes. Within a family, individual greebles shared a body shape but differed in the shape of their four features. Features’ positions were approximately the same for all greebles. Each greeble was matched with an individual label (four letter neologisms starting with a consonant), and each family with a family label (four letter neologisms starting with a vowel). These were the greebles used for the training.

#### Greeble test set

In addition to 25 of the 30 training set greebles (five per family), the test set consisted of ten distorted variants and six morphed variants. Ten training set greebles (two individuals per family) were used to create configurally distorted variants by changing the position of three of the four of the greebles’ attached body parts. Only the body parts’ relative positions and angles were changed while the body parts themselves and the greeble bodies remained unedited, to create distortions on a configural level rather than on a featural level. All distorted greebles were edited in the same manner and the changes will be reported in degrees and percentages of the greebles’ total size, and the changes are visualized in S1 Fig: The upper right body part (P1 in S1 Fig) was placed 30% downwards on the body and rotated by about 45° upwards. The upper left body part (P2 in S1 Fig) was mirrored on the vertical axis and placed about 10% to the right and 10% upward, around the centre of the greeble head. Finally, the leftmost body part (P3 in S1 Fig) was positioned 10% to the right and 15% upwards towards the “neck” area of the body, and angled by 30° upwards. S2 Fig depicts examples of one distorted greeble per family compared to the undistorted variant. Distortions were created using Photoshop CS6®, using 2D depictions of the greebles.

Finally, six averaged greebles (one per family, one across all greebles) were created by morphing a pair of the normal greebles of each family, and morphing the result with the morph between a different pair of greebles of the same family. The morphing result was then morphed with the final member of the family with a 80:20 weighting to create the family average. Finally, pairs of family averages were morphed in the same manner to create a total averaged greeble. 70–100 morphing landmarks were used for each morphing procedure. Landmarks were positioned around greebles’ main bodies, heads, and body parts, as well as along lines indicating the shapes of certain features (e.g., lower body portions which were present in some greebles). Example morphing landmarks of different family averages landmarks are shown in S3 Fig.

**Fig 1:**
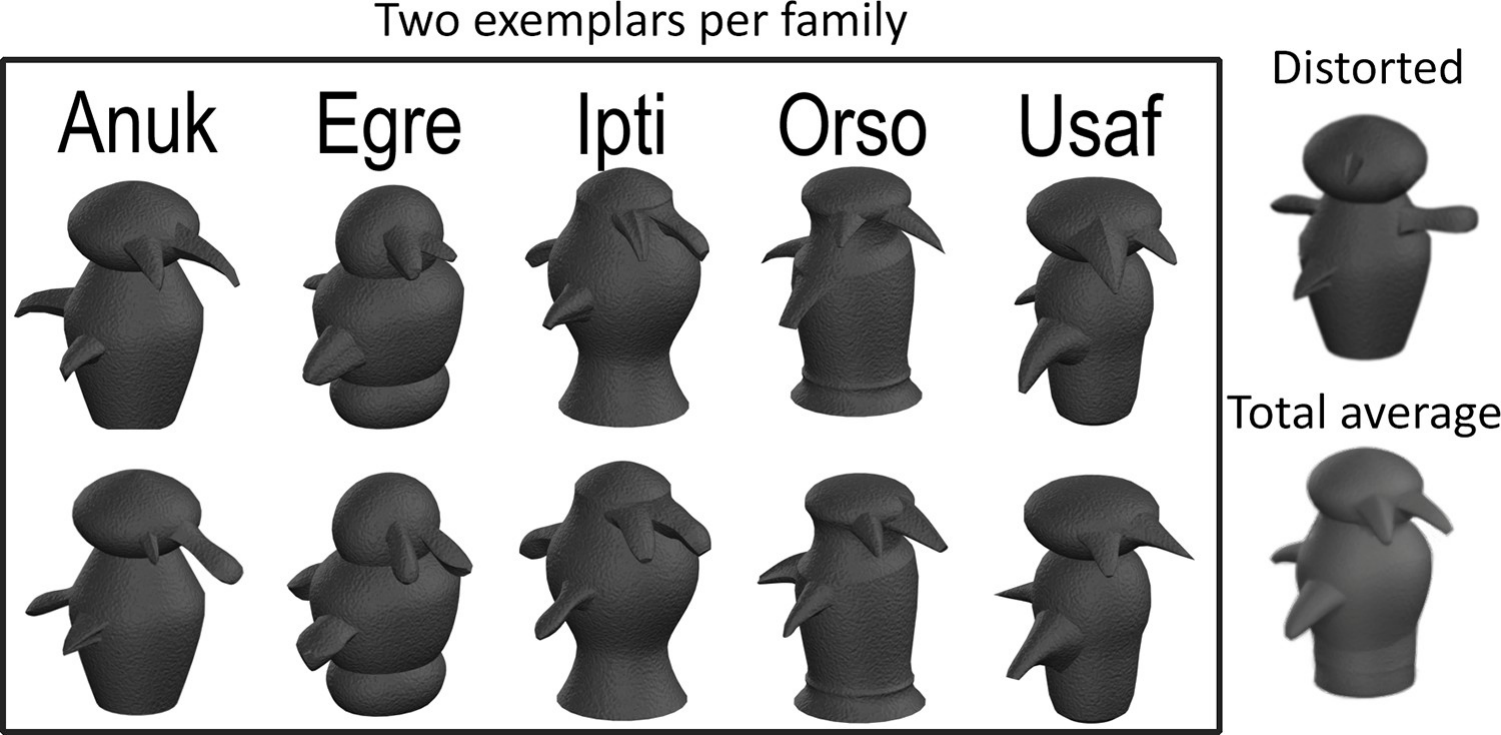
Two individual greebles per family, a distorted greeble of the first family, and the total average. *Note*. Unedited stimulus images courtesy of Michael J. Tarr, Carnegie Mellon University, http://www.tarrlab.org/.

A summary of the greeble families and differences between greeble conditions are depicted in Fig 1. Morphing was conducted via Fantamorph Deluxe®, and morphing noise was eliminated with Photoshop CS6®. All greeble stimuli used can be found in the supplementary files.

### Procedure

#### Expertise training

Expertise training was based on a five-session setup successfully used in previous studies [28, 64]. Only participants within the *training group* completed the expertise training. The different tasks are described below while the procedure is summarized in S1 Table. The training regime took place over five days and took 60 minutes each day on average. The tasks were used to familiarise the participants with the greebles as follows: *Family examples* (2 greebles per family are presented together with the family labels in Fig 1); *Family viewing* (participants view an individual greeble with the respective family label); *Family naming* (participants view an individual greeble and must press the first letter of the greeble’s family name), *Individual viewing* (participants view an individual greeble with the respective individual label), *individual naming* (participants view an individual greeble and must press the first letter of the greeble’s individual name), *Individual naming with feedback* (like *Individual naming* but participants see the correct label after an incorrect response), *Verification* (participants view an individual greeble followed by either a family or individual label that is correct 50% of the time. Participants press *y* when the label is correct, *n* when it is incorrect, and *space* if the greeble or label have not yet been shown before), and *Final verification* (same as verification; the accuracy data was used to check whether participants acquired expertise). Each task had a fixed number of trials which are summarized in S1 Table.

#### Rating task

The rating task was completed by participants of both conditions. *Training condition* participants completed the ratings task after the final expertise session whereas the *control condition* participants had not previously seen any greebles when completing the rating task. Participants rated normal, averaged, and distorted greebles on seven scales ranging from 0 to 100: *eerie*, *creepy*, *strange*, *weird*, *pleasant*, *attractive*, and *appealing*. Based on previous research on the categorization of measures of the uncanny valley [31], *eerie* and *creepy* are combined to an *uncanniness* index, *strange* and *weird* to an abnormality index, and *pleasant*, *attractive*, and *appealing* to an *attractiveness* index. *Uncanniness* thus reflects a negative specific emotional reaction, *abnormality* a judgment of the stimulus’ atypicality or unusualness, and *attractiveness* the stimulus’ aesthetic appeal, all which are related to an uncanny valley [31]. Participants rated a total of 41 greebles (25 normal greebles, 10 distorted greebles, 6 average greebles).

### Analysis, ethics statement, and data availability

Data preparation, data cleaning, and statistical analysis were conducted via JASP and R. Main analysis and post-hoc tests were done via Bayesian mixed-effects analyses of variance (ANOVA) in JASP. After sampling a first set of participants, BF >3 was used as a threshold for the Bayesian stopping rule. Bayesian mixed-level ANOVAs were all conducted with the same default options on JASP (Prior r scale fixed effects = 0.5, prior r scale random effects = 1. Further specifications to reproduce the results are available on OSF: https://osf.io/zsnkr/). Fixed and random effects were defined for each analysis (see openly available source code for JASP for how formulas are defined; [65]). Accompanying non-Bayesian ANOVAs and post-hoc tests were done with R, and linear mixed models were used for post-hoc tests. Linear mixed models produce large degrees of freedom seen in the results section [66, 67]. R packages *ez* (function *ezANOVA()*)and *nlme* (function *lme()*)were used for ANOVAs and linear mixed models, respectively.

The study was approved by the Cardiff University School of Psychology Research Ethics Committee in March 2021 (reference number: EC.21.02.09.6291R). The data and R code for the analysis is available at: https://osf.io/zsnkr/.

Evaluation of the results is based on the size of the BF as recommended [68, 69]: BF_10_ < 1 is interpreted as no evidence for the alternative hypothesis, 1 < BF_10_ < 3 as weak evidence, 3 < BF_10_ < 10 as moderate, and BF_10_ > 10 as strong evidence. BF have been prioritized over p-values in evaluating one hypothesis over another, as there are limitations in interpreting significance of p-values [70].

## Results

### Expertise acquisition

Because an accuracy of 33% in the final verification chance would indicate a random chance response, only trained participants with an accuracy above 40% were included in the analysis. The 40% threshold was selected to exclude the potential of random chance responses. One of the 21 participants in the expertise condition had an accuracy below the threshold (35%) and was thus excluded from the analysis. The remaining participants’ average accuracy in the final verification task was *M* = 70.29%, *SD* = 14.94%.

### Greeble rating

#### Rating scales

The items *eerie* and *creepy* were combined into an *uncanniness* index, the items *strange* and *weird* into an *abnormality* index, and the items *pleasant*, *attractive*, and *appealing* into an *attractiveness* index. Indices were averages of the items across data. Outlier values of *uncanniness* and *attractiveness*, defined as +/- 1.5 of the interquartile range from the median, were detected and removed for all 2x5 conditions (three outlier values in total). Across all participants, *uncanniness* had a Cronbach’s alpha of *α* = .876, *abnormality* had *α* = .816, and *attractiveness α* = .868, indicating good reliability for all three indices. The scales’ intercorrelations are summarized in S2 Table.

#### Uncanniness and abnormality ratings

**Fig 2:**
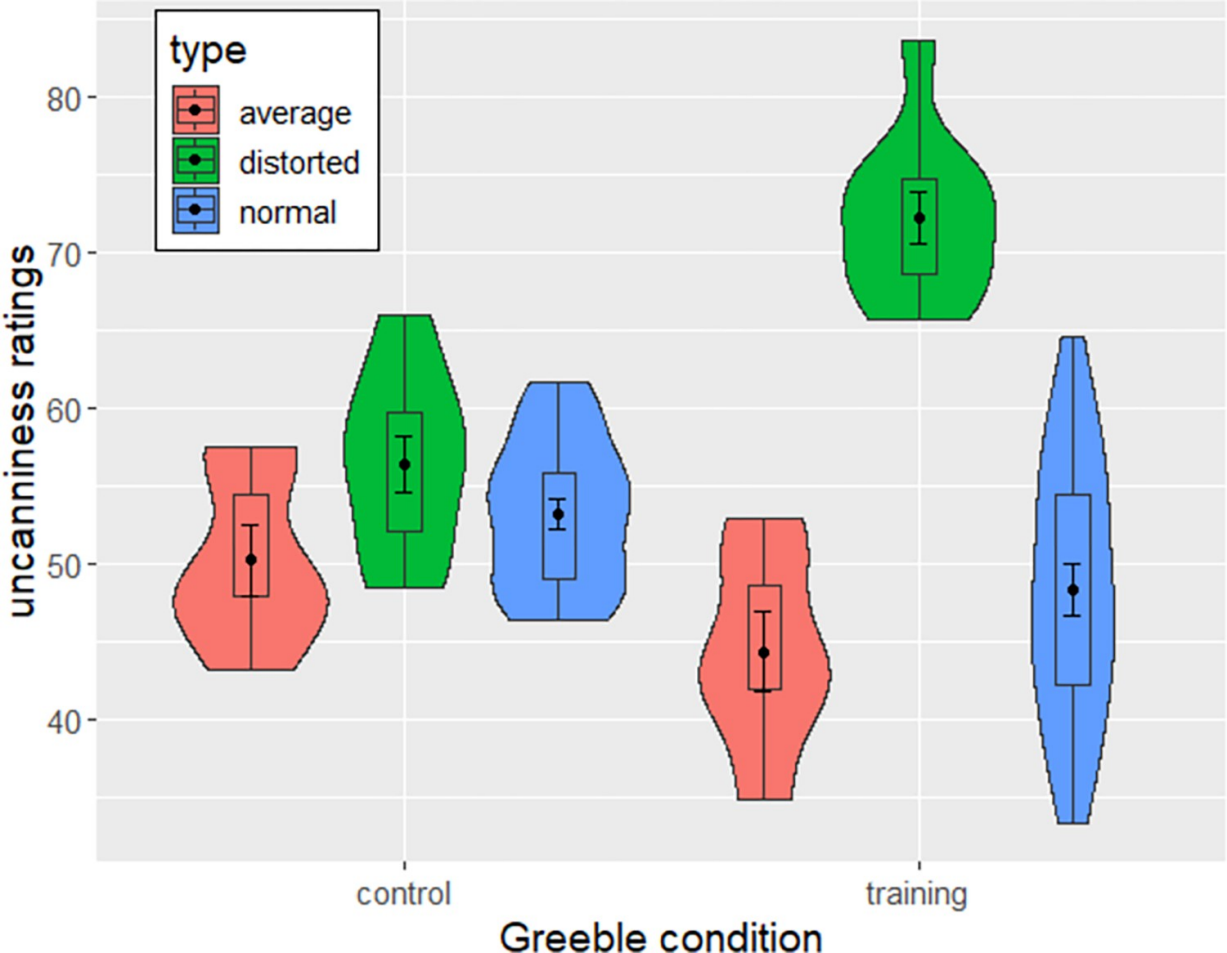
Violin and boxplots depicting uncanniness ratings across greeble types and conditions. Error bars show standard errors.

The mean uncanniness ratings for each type of greeble for expertise and control participants are shown in Fig 2. A mixed-effects ANOVA with greeble type (average, normal, or distorted) and condition (training or control) as predictors of uncanniness ratings and participants as within-subject variables showed strong evidence for a main effect of greeble type (BF_10_ = 1206.401, *F*(2, 80) = 34.528, *p* < .001, *η*^*2*^ = .16) but no main effect of condition (BF_10_ = 0.307, *F*(1, 40) = 0.086, *p* = .770, *η*^*2*^ < .01). However, there was strong evidence for an interaction between type and condition (BF_10_ = 4.101e^12^, *F*(2, 80) = 19.559, *p* < .001, *η*^*2*^ = .09) on uncanniness ratings. The same pattern was observed for abnormality ratings (main effect type: BF_10_ = 508.565, *F*(2,80) = 35.067, *p* < .001, *η*^*2*^ = .11; main effect condition: BF_10_ = 0.361, *F*(1,40) = 0.124, *p* = .726, *η*^*2*^ < .01; interaction condition and type: BF_10_ = 7.598e^11^, *F*(2,80) = 15.471, *p* < .001, *η*^*2*^ = .05).

P-adjusted post-hoc Tukey tests were conducted to further investigate the interaction between greeble type and condition. Linear mixed models were used for non-Bayesian post-hoc tests, with greeble types and condition as fixed effects and participants as random effects. Comparisons showed no evidence in favor of distorted greebles being more uncanny than normal greebles without the training (BF_10_ = 0.279, *t* (1501) = 1.711, *p*_adj_ = .488, *d* = .14), there was strong evidence that distorted greebles were more uncanny than normal greebles after expertise training (BF_10_ = 1.894e^20^, *t*(1501) = 13.399, *p*_adj_ < .001, *d* = 1.2). Most interestingly, there was strong evidence that distorted greebles were more uncanny after training than distorted greebles in the control condition (BF_10_ = 20959.55, *t*(1501) = 2.909, *p*_adj_ = .027, *d* = 0.66).

Finally, there was no evidence that averaged greebles were less uncanny than normal greebles in the control condition (BF_10_ = 0.168, *t*(1501) =, *p*_adj_ = .311) nor in the training condition (BF_10_ = 0.366, *t*(1501) = -1.431, *p*_adj_ = .153).

**Fig 3:**
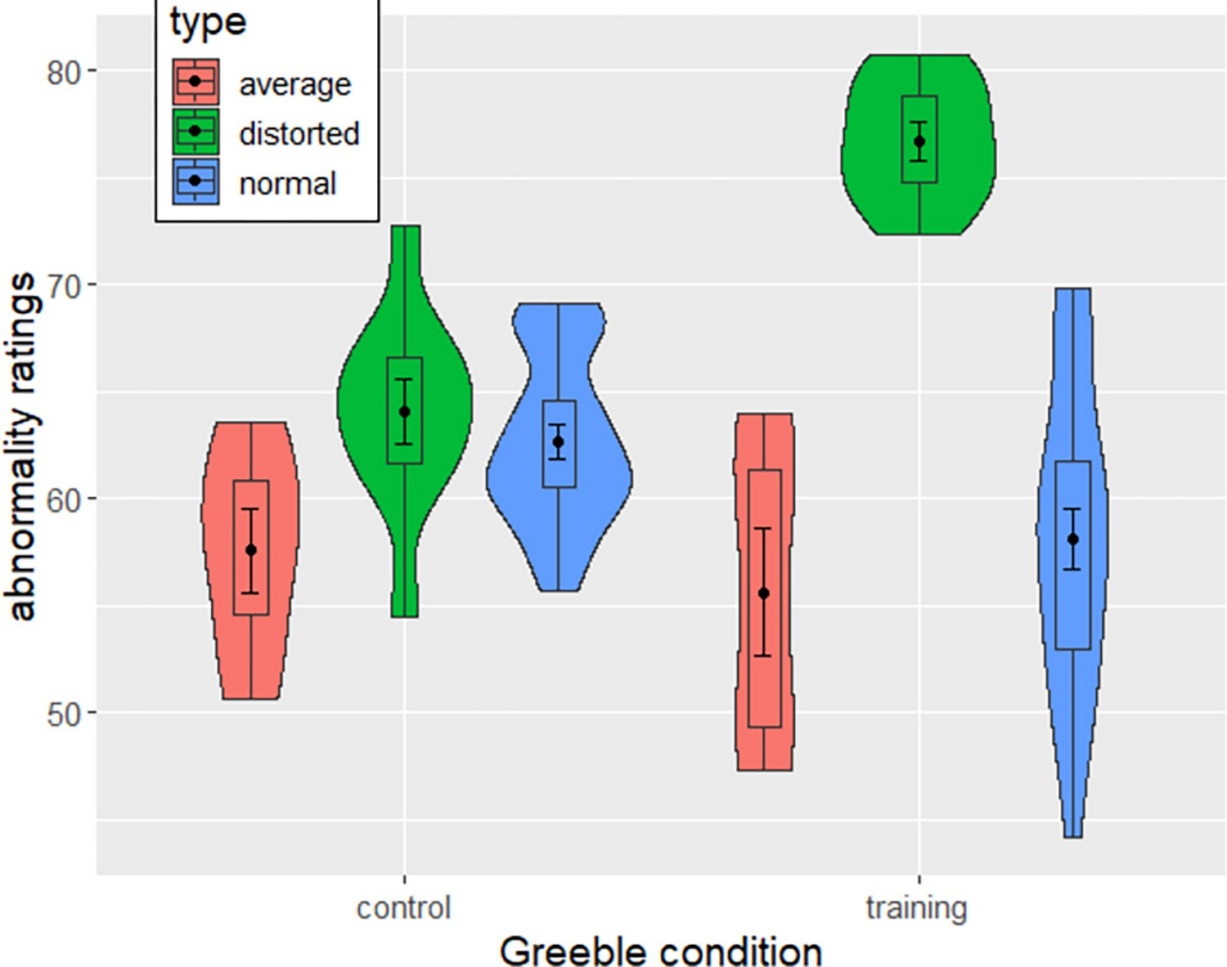
Violin and boxplots depicting abnormality ratings across greeble types and conditions. Error bars show standard errors.

Abnormality ratings follow a similar pattern with strong evidence in favor of the model: Distorted greebles were not more abnormal than normal greebles without training (BF_10_ = 0.12, *t*(1501) = 0.719, *p*_adj_ = .98), but they were more abnormal after the training with strong evidence (BF_10_ = 5.363e^12^, *t*(1501) = 11.519, *p*_adj_ < .001, *d* = 1.03). In addition, there was strong evidence that distorted greebles were more abnormal after training than without training (BF_10_ = 78.266, *t*(1501) = 1.875, *p*_adj_ = .372). Ratings are depicted in Fig 3.

#### Attractiveness ratings

**Fig 4:**
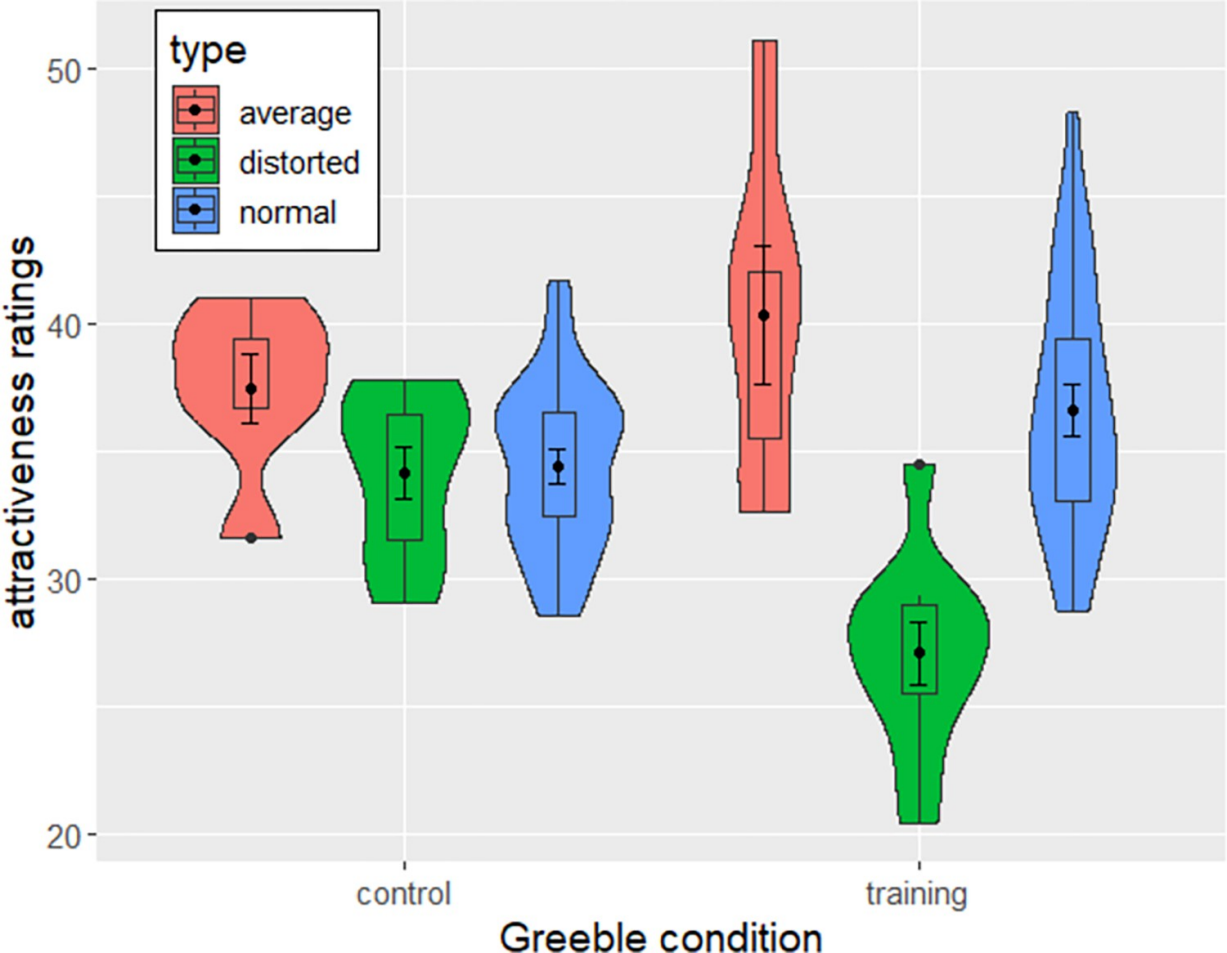
Violin and boxplots depicting attractiveness ratings across greeble types. Error bars show standard errors.

Fig 4 depicts the mean *attractiveness* ratings for the three greeble types of the expertise and control conditions. Similarly to *uncanny* ratings, the ANOVA with greeble type and condition as predictors of *attractiveness* ratings showed moderate evidence for a main effect of greeble type (BF_10_ = 4.46, *F*(2, 80) = 25.184, *p* < .001, *η*^*2*^ = .06) but not for a main effect of condition (BF_10_ = 0.299, *F*(1, 40) = 0.03, *p* = .861, *η*^*2*^ = .00), and strong evidence for an interaction effect between greeble type and condition (BF_10_ = 6705.494, *F*(2, 80) = 8.707, *p* < .001, *η*^*2*^ = .02) on *attractiveness* ratings.

Post-hoc Tukey tests furthermore showed no evidence that average greebles were more attractive than normal greebles in either the control condition (BF_10_ = 0.334, *t*(1463) = 2.055, *p*_*adj*_ = .04, *d* = 0.225), or after expertise training (BF_10_ = 0.398, *t*(1463) = 2.164, *p*_*adj*_ = .043, *d* = 0.15). However, the p-values indicated significance in both tests. Furthermore, there was no evidence that distorted greebles were less attractive than normal greebles in the control condition (BF_10_ = 0.096, *t*(1463) = 0.732, *p* = .46, *d* = .16), but strong evidence in the training condition (BF_10_ = 52297.5, *t*(1463) = -7.516, *p* < .001, *d* = .68).

### Verification task accuracy as a predictor

Given the prediction that expertise modulates the effect of deviation on uncanniness, a higher degree of expertise would likely predict stronger uncanniness (and abnormality) and lower appeal ratings of distorted greebles. Thus, post-hoc regression model analyses were conducted with verification task accuracy as a predictor for uncanniness, abnormality, and appeal ratings respectively, in the training condition. Results indicate no evidence for verification accuracy as a predictor for uncanniness (BF_10_ = 0.215, *t*(633) = -1.566, *p* = .118, *R*^2^ = 0.003), moderate evidence for predicting abnormality (BF_10_ = 3.804, *t*(633) = 2.932, *p* = .004, *R*^2^ = .011), and weak evidence for predicting appeal (BF_10_ = 1.097, *t*(633) = 2.334, *p* = .02, *R*^2^ = .008). Thus, verification accuracy, a proxy for the degree of acquired expertise, slightly predicted aesthetic ratings of greebles.

### Changes of ratings across trials

While the main analysis provides insight into the effect of expertise on uncanniness, abnormality, and attractiveness ratings of deviating and prototypical variants, it is not clear whether expertise specifically, or a different process like general familiarization or exposure, is necessary to facilitate the difference between greeble types. In addition, according to p-values (but not Bayesian statistics), prototypical greebles were more attractive than normal greebles without expertise training. This difference may be the result of a prototypicality preference after early exposure (and thus an increase of attractiveness of averaged greebles across trials), or because of characteristics intrinsic to the stimuli (e.g., morphing artefacts). To investigate whether early expose affects the ratings of greebles (e.g., an early increase of average greebles by the end of the rating task), post-hoc analyses on the greeble type ratings were conducted depending on the greebles’ trial occurrence, for both the control and training condition. Thus, the post-hoc analysis aims to investigate an effect of early familiarity on changes in ratings of greeble types by testing for an interaction between greeble types and trial number.

Linear mixed models were used to investigate the effect of the trial on which the stimulus occurred on ratings, across greeble types and conditions, with trial, type, and condition as fixed effects and participants as random effects. However, no interaction with trials was significant for uncanniness (trial and type: BF_10_ = 2.910e^-4^, *t*(1414) = -1.46, *p* = .887; trial and condition: BF_10_ = 6.786e^-6^, *t*(1414) = -1.36, *p* = .144; trial, type, and condition: BF_10_ = 1.244e^-9^, *t*(1414) = 0.35, *p* = .726), abnormality (trial and type: BF_10_ = 5.262e^-6^, *t*(1414) = -0.309, *p* = .757; trial and condition: BF_10_ = 8.338e^-6^, *t*(1414) = -0.038, *p* = .97; trial, type, and condition: BF_10_ = 0.003, *t*(1414) = -0.416, *p* = .678), or attractiveness (trial and type: BF_10_ = 1.047e^-6^, *t*(1414) = -0.143, *p* = .887; trial and condition: BF_10_ = 5.585e^-6^, *t*(1414) = -0.196, *p* = .845; trial, type, and condition: BF_10_ = 5.022e^-9^, *t*(1414) = 0.639, *p* = .523).

## Discussion

Greebles are a set of novel objects used here to manipulate expertise acquisition [26]. Thus, they are a useful tool to assess the effect of expertise on the aesthetic evaluation of structurally deviating objects. Here it was shown that greeble experts and greeble non-experts evaluate the uncanniness and attractiveness of deviating greebles differently.

### The deviation-from-familiarity effect

In line with the hypothesis, the results show that distorted greebles were significantly more uncanny than normal greebles after expertise training, but not in the control condition. Furthermore, post-training distorted greebles were significantly more uncanny than distorted greebles without expertise training. Thus, expertise increased the uncanniness of deviating exemplars. This *deviation-from-familiarity effect* (increased uncanniness of stimuli that deviate from a highly familiar norm) provides insight into negative evaluation of distorted exemplars, and into the uncanny valley specifically: Uncanniness of humanlike and similar entities may not be explained by mechanisms specific to certain categories of stimuli like humans and animals, such as disease avoidance, mind perception, or dehumanization, but could also occur *specifically* due to a deviation from categories of stimuli that humans have familiarized over the course of (a part of) their life, like faces and facial expressions, bodies, voices, and biological motion. The results complement previous research showing that changes in uncanniness ratings are more sensitive to distortions in familiar categories like human compared to animal faces [6, 7], realistic compared to unrealistic faces [4, 5, 40], one’s own face compared to a stranger’s face [71], and familiar compared to unfamiliar and upright compared to inverted faces [40]. In general, the deviation from familiarity effect predicts a generality of the uncanny valley phenomenon (or uncanniness) beyond previous suggestions of human or animal specificity [2, 33, 39], and could extend to inanimate yet familiar categories like written text or physical places, which could be explored in future research.

### Uncanniness and animacy

Some theories on the uncanny valley explain uncanniness through changes in animacy perception [10, 34–37]: stimuli may be uncanny because they straddle boundaries of animacy perception, or because they are “dehumanized” through a subtraction of animacy perception. Past research associated greeble expertise training with animacy [72], and it has been argued that greebles already look animate [73]. Animacy was not measured in this study. Distorted post-expertise greebles as used here may have been uncanny because they approximated post-expertise normal greebles (which could be perceived as animate), yet deviated from them and thus were either perceived as ambiguously animate, or were subtracted animacy. In this sense, uncanniness may not be the result of deviation from familiarity per se, rather than from anomalies in animacy attribution (which, in turn, would result from deviations from familiar patterns). Alternatively, greebles may be perceived as animate regardless of expertise level, and animacy combined with deviation may elicit uncanniness.

### Deviation from familiarity in simpler patterns

The greeble stimuli used in this research are complex stimuli and have been criticized as too animate or animal-like [73]. A similarity to animal appearance may have been an important factor in the observed results, and the reason deviating greebles were uncanny in this study may have been because of their animal-likeness. The deviation from familiarity hypothesis however would predict that sufficient familiarity or expertise with *any kind* of category would make deviating exemplars appear uncanny, potentially including simple patterns like checker boards or gabor patches. In addition, it is unclear whether it is a general familiarity (experience with a variety of exemplars within a category) which drives the observed effect, or whether individuation at a sub-categorical level (expertise) is necessary.

### Attractiveness and deviation

Similar to the pattern observed for *uncanniness* ratings, distorted greebles were less attractive than normal greebles but only on the training condition. Thus, the *deviation-from-familiarity effect* is not specific to uncanniness but can be applied to negative aesthetic evaluations in general, beyond experiences of uncanniness (e.g., ugliness). A negative correlation between uncanniness and attractiveness or likability has been demonstrated in previous research [33, 74]. Experience of negative affect may decrease perceived attractiveness or likability of an artificial entity, but so can structural deviation: Ugly and Botox (and thus highly distinctive) faces are more creepy than normal faces [75]. Thus, evaluations of attractiveness and uncanniness may have similar underlying processes of deviation detection.

One approach to decorrelate the negative association between ratings of attractiveness and uncanniness is by using stimuli that are both attractive and uncanny: For example, sex dolls or sex robots are anecdotally uncanny or creepy and simultaneously sold because of their sexual appeal. Exaggerated sexual features and averaged faces that coincide with human deviation, like a lack of facial details and rigid poses and social responses, may thus elicit both sexually attracting and uncanny reactions. In that case, effects of prototypicality and deviation may be combined across multiple dimensions and modalities, where prototypicality in one dimension and deviation in another can elicit a mix of attractiveness and uncanniness, implying that those constructs can be distinguished.

### Attractiveness and prototypicality

Contrary to prediction, averaged greebles were not more attractive than normal greebles regardless of condition according to the Bayesian analysis. A significant difference in attractiveness ratings between normal and averaged greebles was found both in the training and the control group, and thus even for participants who had no previous exposure to greebles. It is possible that a small increase of attractiveness emerged early on for averaged greebles; an early-onset *prototypicality effect* was found in previous research using novel stimuli: Winkielman et al. [76] had participants rate the attractiveness of patterns of dots or shapes shown after a short training and found that the more attractive patterns or shapes were those closer to the (not shown) category’s prototype. Maybe prototypicality (and a general idea of the distribution of exemplar appearances) is established in very early stages of category formation, and a preference of vague familiarity starts to develop as soon as multiple novel stimuli are experienced for the first time. Thus, an exposure-related mechanism could have been already active without expertise or prolonged experience [77].

However, post-hoc investigations showed that attractiveness ratings of average greebles did not increase more than those of normal greebles in the control condition, although this would be expected in an early onset *prototypicality effect*. Instead, the data indicates that the difference between averaged and normal greebles’ attractiveness ratings was constant across trials, even when the greebles were presented within the first few trials. This initial difference cannot be explained by a prototypicality preference, and instead is likely due to characteristics intrinsic to the averaged models. One potential morphing artefact increasing the attractiveness of averaged greebles is “surface smoothness”, as morphing eliminated the greebles’ surface pattern. Alternatively, morphing could have reduced certain exaggerated features of individual greebles like angular or especially curved shapes, or very small or big features relative to the bodies, which may have been perceived as less appealing. In total, the results speak against an effect of prototypicality preference in greebles.

### Why are deviating exemplars uncanny?

Repeated view of normal greebles during the training could have increased their positive evaluation while not affecting the rating of unfamiliar, distorted greebles [77]. Mere exposure, specifically a lack of exposure for uncanny stimuli, has been proposed as an explanation of the uncanny valley [78]. Increased exposure of multiple stimuli that are grouped based on structural similarity could lead to *blending* and thus a preference to prototypicality [79]. However, training also *increased* the uncanniness ratings of distorted greebles despite their similarity to normal greebles. Thus, the observed *deviation-from-familiarity effect* cannot be explained by a mere exposure effect of non-deviating stimuli.

Expertise strengthens the ability to detect differences between individual exemplars. Thus, hypothetically, expertise could have amplified small pre-existing rating biases between greebles, rather than facilitating a normal categorical variation (e.g., distorted greebles could have been slightly, but not significantly, more uncanny than normal greebles even without expertise due to factors like morphing noise. Expertise would then enhance the uncanniness difference between greeble types). However, a small attractiveness bias towards averaged greebles is present both without and with expertise training with comparable effect sizes (*d* = 0.225 and *d* = 0.15). Furthermore, distorted greebles did not differ from normal greebles in the control condition on any rating, but they did in the training condition. Thus, the data do not indicate any biases that have been amplified by expertise training.

Novelty avoidance proposes that stimuli are uncanny because they are novel [63]. While distorted greebles were presented to the training condition participants for the first time and thus would be relatively more novel than the normal greebles, post-training distorted greebles are still *less* novel than distorted greebles in the control condition. Despite being more familiar, post-training distorted greebles were less uncanny than in the control condition. In addition, averaged greebles were also presented for the first time, but were not more uncanny than normal greebles in the training condition. Thus, novel greebles were not automatically uncanny, and deviation from the familiar variance can explain the results better than novelty avoidance. In general, it seems that the proximity of a novel stimulus to a familiar pattern is integral to the observed effect: deviating greebles were only uncanny after a normal variation of greebles has been experienced. This deviation from familiarity effect could be explained by cognitive disfluency, as the distance between a familiar pattern and the deviating variant could elicit disfluent processing. Alternatively, the discrepancy between a learnt pattern and a deviating exemplar could elicit a prediction error, which has been proposed to underlie the uncanny valley in previous research [80]: The recognition of the general shape of a greeble could create a mental predictive model of the greeble after training; however, the deviating features would then violate the more specific predictions of the greeble’s appearance (i.e., the position of the body parts), and elicit discomfort.

### Further questions

This study’s results raise multiple questions for future research. First, distorted greebles in this study always consisted of the same pattern of distorted configuration. However, distortion can vary greatly both quantitatively (degree of distortion across one dimension) and qualitatively (distortions across different dimensions). Future research can, for example, investigate how the degree of distortion influences uncanniness ratings. Similarly, it would be interesting to see how the amount of experience influences the sensitivity to distortions: At what point during the expertise training does the *deviation-from-familiarity effect* (and the preference for prototypicality) occur, does it increase with prolonged experience, and does it get more sensitive for subtler distortions? A post-hoc analysis found that verification accuracy (a proxy for acquired expertise) did not significantly predict the greeble uncanniness ratings, but did predict their abnormality and attractiveness ratings, indicating that the acquired level of expertise does influence aesthetic ratings. A potential mechanism is that a higher level of expertise makes deviations from prototypical appearances more apparent [40] leading to negatively experienced cognitive disfluency [54]. Future research can investigate the effect of the acquired level of expertise on aesthetic ratings of deviating variants more thoroughly, for example by manipulating the duration and intensity of expertise training.

Second, investigating neural correlates of the *deviation-from-familiarity effect* and its development is of interest to better understanding the effect. According to the cognitive fluency theory, distorted greebles should elicit stronger activation patterns than normal greebles in greeble-selective brain areas after expertise training. Furthermore, it would be interesting to investigate whether post-training distorted greebles elicit prediction errors like those observed in prior research on the uncanny valley [80].

Third, this study did only investigate the affective and aesthetical judgment of the greebles, neglecting possible cognitive components. Future research can look at cognitive mechanisms underlying the processing of greeble stimuli like categorization difficulty [41–43], distortion sensitivity [40], configural processing [6], and whether expertise itself is necessary for the *deviation-from-familiarity effect* or if prolonged experience alone is sufficient.

Fourth, as the goal of this study was to investigate the effect of deviation and expertise on uncanniness *in principle*, only one distortion type and degree was used. Further research can investigate the interaction between the degree of deviation or distortion, and expertise, as well as the type of distortion. Previous research has shown that in faces, increasing distortions are perceived as increasingly more uncanny [40]; a similar relationship may be predicted for greebles.

Fifth, this study did not measure the perception of animacy of greebles. As animacy has been both associated with the uncanny valley [37] and greebles [72, 73], the observed effect of deviation from familiarity on uncanniness could potentially be mediated by animacy. Future research can investigate the role of animacy by measuring animacy perception or by using more object-like (compared to potentially animal-like) stimulus sets.

Finally, while it is suggested here that the observed difference between distorted and normal greebles would correspond to the portion in Mori’s [2] graph after the valley drop into uncanniness towards full human likeness (analogous to how distorted yet realistic faces would fall into the valley and then increase towards full human likeness with decreasing distortion). However, this study did not replicate a “proper” uncanny valley curve. The observed effects are similar to those found by Diel & Lewis [40] using face stimuli, which could be plotted along an uncanny valley function, finding that with increasing face realism, the sensitivity to distortion increased. Future research could attempt to replicate an uncanny valley of greeble by using greebles of different realism levels (e.g., normal greebles and abstract drawings of greebles).

## Conclusion

This study observed an experience-dependent *deviation-from-familiarity effect*: structurally deviating exemplars are perceived as uncanny after familiarisation compared to the same stimuli without sufficient experience with normal variants. Thus, this study is the first in testing the effect of expertise on uncanniness and attractiveness ratings of deviating and averaged stimuli. The results provide evidence for a general, experience-dependent mechanism underlying the development of uncanniness, specifically that increased expertise with a category sensitizes the negative evaluation of structurally deviating exemplars. The results carry implications for the uncanny valley and further topics on the effect of experience on negative aesthetic judgments.

## Supporting information

S1 Table Experiment procedure for both control and training group. Numbers represent number of trials and numbers in brackets represent number of individual greebles the participant has been introduced to before in an "individual viewing" task, and a number in brackets plus “new” indicates the number of new greebles shown. “Rating” refers to either the control or post-training rating session.
(DOCX)

S2 Table Intercorrelations of the scales used in the experiment. (DOCX)

S1 Fig Distortion procedure visualized on a single (Anuk family) greeble example. The same procedure was used for every distorted variant.
(TIF)

S2 Fig One distorted greeble per family (upper row) and its normal variant (lower row). The same distortion principle was used for each greeble.
(TIF)

S3 Fig Morphing landmarks for the total average morphing in FantaMorph Deluxe. Pairs of greebles were morphed together, here the morphed averages of family 1 (left) and 2 (right). Afterwards, the result was morphed with the morph between the averages of family 3 and 4, and finally with the average of family 5 with an 80:20 weighting to create a total average. After each morphing procedure morph noise was cleaned using Photoshop CS6.
(TIF)
