# Supplementary material for: The deviation-from-familiarity effect: Expertise increases uncanniness of deviating exemplars
Source: PLoS One. 2022 Sep 1;17(9):e0273861. doi: 10.1371/journal.pone.0273861 (PMC9436138; doi:10.1371/journal.pone.0273861)
Supplement: S1 Table — Numbers represent number of trials and numbers in brackets represent number of individual greebles the participant has been introduced to before in an "individual viewing" task, and a number in brackets plus “new” indicates the number of new greebles shown. “Rating” refers to either the control or post-training rating session. (DOCX) [file pone.0273861.s001.docx]

| Procedure (number of individual greebles shown) | Session 1 | | Session 2 | Session 3 | | Session 4 | Rating | |
| --- | --- | --- | --- | --- | --- | --- | --- | --- |
| Family examples (10) | | 1 |  |  |  | |  |  |
| Family viewing (25) | | 25 |  |  |  | |  |  |
| Family naming (30) | | 30 |  |  |  | |  |  |
| Individual viewing (5) | | 20 (5) |  |  |  | |  |  |
| Individual naming with feedback (5) | | 15 (5) |  |  |  | |  |  |
| Individual naming (30) | | 60 (5) |  |  |  | |  |  |
| Verification (30) | | 125 (5) |  |  |  | |  |  |
| Family naming (30) | | 30 |  |  |  | |  |  |
| Individual viewing  (previously learned) | | 10 (5) | 20 (5) | 40 (10) | 60 (15) | |  |  |
| Individual naming (30) | | 60 (5) |  |  |  | |  |  |
| Verification (30) | | 125 (5) | 125 (5) | 130 (10) | 120 (15) | |  |  |
| Individual viewing (5) | |  | 20 (5 new) | 20 (5 new) | 20 (5 new) | |  |  |
| Individual naming with feedback (previously learned) | |  | 30 (10) | 45 (15) | 60 (20) | |  |  |
| Individual naming (30) | |  |  | 60 (15) | 60 (20) | |  |  |
| Verification (30) | |  | 130 (10) | 125 (15) | 120 (20) | |  |  |
| Individual naming (30) | |  | 60 (10) | 60 (15) | 60 (20) | |  |  |
| Verification (30) | |  | 130 (10) | 125 (15) | 120 (20) | |  |  |
| Individual naming (30) | |  | 60 (10) | 60 (15) | 60 (20) | |  |  |
| Final verficiation | |  |  |  | 120 (20) | |  |  |
| Rating task (41) | |  |  |  |  | | 41 |  |
