# Supplementary material for: The deviation-from-familiarity effect: Expertise increases uncanniness of deviating exemplars
Source: PLoS One. 2022 Sep 1;17(9):e0273861. doi: 10.1371/journal.pone.0273861 (PMC9436138; doi:10.1371/journal.pone.0273861)
Supplement: S2 Table — (DOCX) [file pone.0273861.s002.docx]

|  |  | Eerie | Creepy | Strange | Weird | Attractive | Pleasant | Appealing |
| --- | --- | --- | --- | --- | --- | --- | --- | --- |
| Eerie |  | 1 |  |  |  |  |  |  |
| Creepy |  | .786 | 1 |  |  |  |  |  |
| Strange |  | .480 | .534 | 1 |  |  |  |  |
| Weird |  | .585 | .600 | .705 | 1 |  |  |  |
| Attractive |  | -.325 | -.333 | -.481 | -.42 | 1 |  |  |
| Pleasant |  | -.448 | -.486 | -.455 | -.529 | .631 | 1 |  |
| Appealing |  | -.363 | -364 | -.504 | -.444 | .755 | .69 | 1 |
