# Supplementary figures and images for: The deviation-from-familiarity effect: Expertise increases uncanniness of deviating exemplars
Source: PLoS One. 2022 Sep 1;17(9):e0273861. doi: 10.1371/journal.pone.0273861 (PMC9436138; doi:10.1371/journal.pone.0273861)

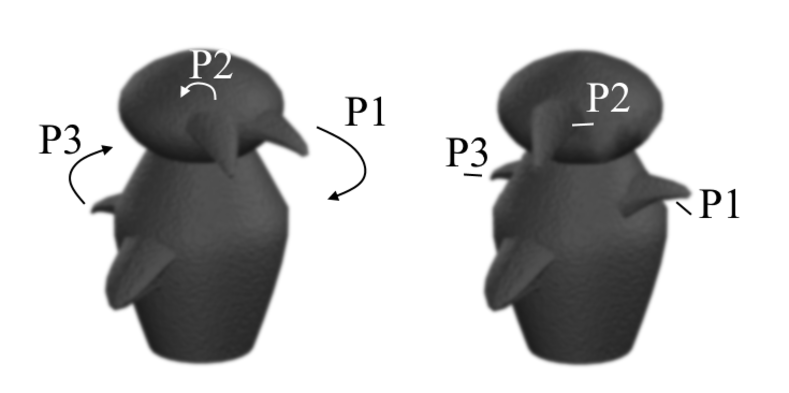

Supplement: S1 Fig — The same procedure was used for every distorted variant. (TIF) [file pone.0273861.s003.tif]

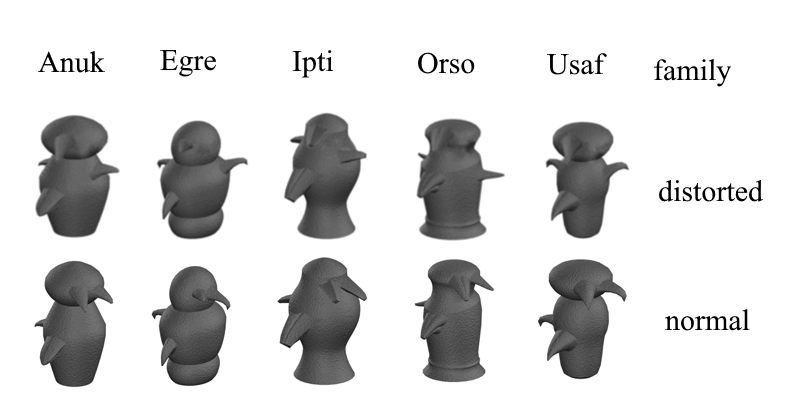

Supplement: S2 Fig — One distorted greeble per family (upper row) and its normal variant (lower row). The same distortion principle was used for each greeble. (TIF) [file pone.0273861.s004.tif]

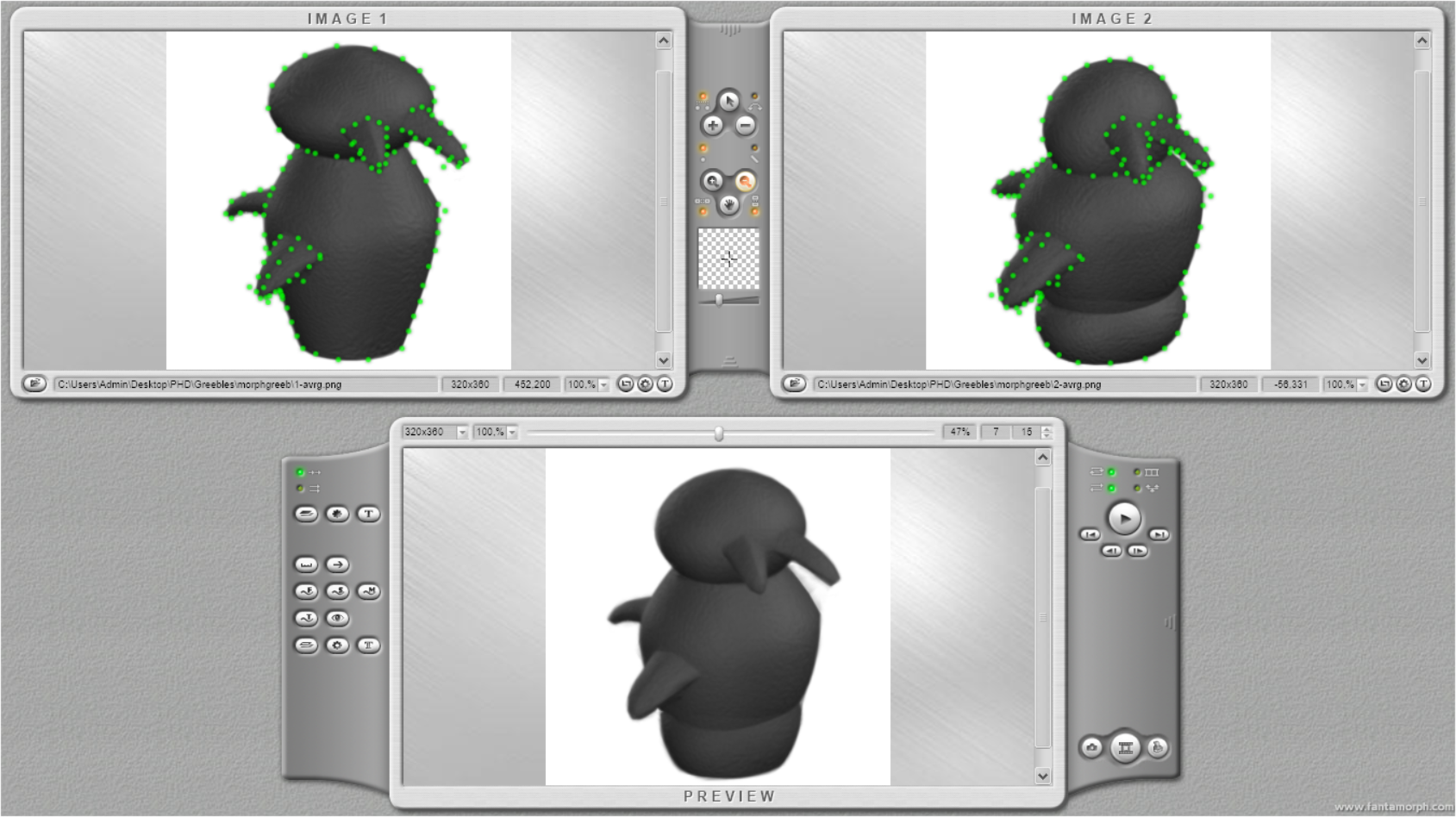

Supplement: S3 Fig — Pairs of greebles were morphed together, here the morphed averages of family 1 (left) and 2 (right). Afterwards, the result was morphed with the morph between the averages of family 3 and 4, and finally with the average of family 5 with an 80:20 weighting to create a total average. After each morphing procedure morph noise was cleaned using Photoshop CS6. (TIF) [file pone.0273861.s005.tif]
